# Steps to a HealthierUS Cooperative Agreement Program: Foundational Elements for Program Evaluation Planning, Implementation, and Use of Findings

**Published:** 2005-12-15

**Authors:** Goldie MacDonald, Danyael Garcia, Stephanie Zaza, Michael Schooley, Don Compton, Terry Bryant, Lulu Bagnol, Cathy Edgerly, Rick Haverkate

**Affiliations:** Centers for Disease Control and Prevention; Centers for Disease Control and Prevention, Atlanta, Ga; Centers for Disease Control and Prevention, Atlanta, Ga; Centers for Disease Control and Prevention, Atlanta, Ga; Centers for Disease Control and Prevention, Atlanta, Ga; Colorado Department of Public Health and Environment, Denver, Colo; Inter-Tribal Council of Michigan, Sault Ste Marie, Mich; Inter-Tribal Council of Michigan, Sault Ste Marie, Mich; Inter-Tribal Council of Michigan, Sault Ste Marie, Mich

## Abstract

The Steps to a HealthierUS Cooperative Agreement Program (Steps Program) enables funded communities to implement chronic disease prevention and health promotion efforts to reduce the burden of diabetes, obesity, asthma, and related risk factors. At both the national and community levels, investment in surveillance and program evaluation is substantial. Public health practitioners engaged in program evaluation planning often identify desired outcomes, related indicators, and data collection methods but may pay only limited attention to an overarching vision for program evaluation among participating sites.

We developed a set of foundational elements to provide a vision of program evaluation that informs the technical decisions made throughout the evaluation process. Given the diversity of activities across the Steps Program and the need for coordination between national- and community-level evaluation efforts, our recommendations to guide program evaluation practice are explicit yet leave room for site-specific context and needs. Staff across the Steps Program must consider these foundational elements to prepare a formal plan for program evaluation. Attention to each element moves the Steps Program closer to well-designed and complementary plans for program evaluation at the national, state, and community levels.

## The Steps to a HealthierUS Cooperative Agreement Program

The Steps to a HealthierUS Cooperative Agreement Program (Steps Program) enables funded communities to implement chronic disease prevention and health promotion efforts to reduce the burden of diabetes, obesity, asthma, and related risk factors — physical inactivity, poor nutrition, and tobacco use. The Steps Program funds communities in three categories: state-coordinated small cities or rural areas, large cities or urban areas, and tribes or tribal entities. In fiscal year 2003, the Department of Health and Human Services (HHS) distributed $13.6 million to 12 programs representing 24 communities (7 large cities, 1 tribe, and 4 states coordinating awards to 16 small cities and rural communities). In fiscal year 2004, HHS distributed $35.8 million to increase support to existing communities and fund an additional 10 programs representing 16 communities (5 large cities, 2 tribes, and 3 states coordinating awards to 9 small cities and rural communities). To date, the Steps Program includes 40 communities nationwide.

In addition to fiscal resources, HHS provides oversight and technical expertise to support evidence-based program planning and implementation, disease and risk factor surveillance, and program evaluation. The allocation of resources to surveillance and evaluation meets the recommended 10% of total program dollars in the majority of funded communities (1,2). Disease and risk factor surveillance is an important source of information for program planning and evaluation at both the national and community levels. Thus, funded communities participate annually in the Behavioral Risk Factor Surveillance System (BRFSS) and biennially in the Youth Risk Behavior Surveillance System (YRBSS). Thus, program evaluation builds upon  surveillance data and includes community-specific efforts to assess program implementation and progress at individual sites. All funded communities participate in coordinated national-level evaluation activities that focus on the Steps Program as a whole. The purpose of national-level program evaluation activities includes the following: assessing the merit or worth of the Steps Program or key efforts; documenting program processes; determining progress toward intended outcomes; demonstrating accountability to diverse stakeholders; and identifying opportunities for ongoing program development and improvement.

## Foundational Elements for Program Evaluation Planning, Implementation, and Use of Findings

Public health practitioners engaged in program evaluation planning often identify a stream of program outcomes, related indicators, and data collection methods but may pay only limited attention to developing an overarching vision for program evaluation among participating sites. Because of the need for coordination between national and community-level evaluation efforts, recommendations to guide program evaluation practice must be explicit; however, they must also be flexible enough to accommodate the diversity of programmatic activities and community-specific needs. It is important to remember that "the term evaluation does not imply a particular type of a study design" (3); the elements described here provide a vision of program evaluation to guide the technical decisions made throughout the evaluation process (e.g., identification of indicators and data collection methods).

We have developed a set of foundational elements for program evaluation planning, implementation, and use of findings to clarify expectations for national and community-specific practice. For the Steps Program, these elements include the following: 1) distinguish between research and program evaluation; 2) define program evaluation; 3) use the *Framework for Program Evaluation in Public Health* to inform evaluation planning; 4) seek cultural competence throughout the evaluation process; 5) prepare a program logic model as a platform for evaluation planning, implementation, and use of findings; 6) identify the purposes of the evaluation; 7) identify intended users and uses of the evaluation; 8) identify key evaluation questions; 9) attend to process and outcome evaluation; and 10) maximize the use of existing surveillance systems for outcome measurement. For the Steps Program, considering these foundational elements is necessary to prepare a formal plan for program evaluation. The Table summarizes the elements and provides a list of resources to clarify practice. Attention to each element moves the Steps Program closer to well-designed and complementary plans for program evaluation at the national, state, and community levels.

### 1. Distinguish between research and program evaluation.

A key misunderstanding about program evaluation is that it must follow an academic or epidemiologic research model. For community-based public health programs, this model is typically difficult to implement, if not impossible or inappropriate. Although often considered the "gold standard" for public health research, the "use of randomized control trials to evaluate health promotion initiatives is, in most cases, inappropriate, misleading and unnecessarily expensive" (2). Research and program evaluation differ in purpose and practice along 10 critical dimensions: planning, decision making, standards, questions, design, data collection, analysis and synthesis, judgments, conclusions, and uses (5). See MacDonald et al for an explanation of these differences (5). The differences between research and program evaluation demonstrate the need to consider a wider range of options in evaluation design. Moreover, familiarity among stakeholders with how research and program evaluation differ provides a common vocabulary for discussing and understanding program evaluation. An appropriate approach to the evaluation of community-based programs includes consideration of a wide range of quantitative and qualitative data collection methods in conjunction with relevant standards for program evaluation practice.

### 2. Define program evaluation.

Definitions of program evaluation vary by field of practice and approach. However, they typically include some reference to the "systematic investigation of the worth or merit of an object" (24). For the Steps Program, program evaluation is defined as "the systematic collection of information about the activities, characteristics, and outcomes of programs to make judgments about the program, improve program effectiveness, and/or inform decisions about future program development" (6). The definition includes assessment of program planning, implementation, and intended outcomes. Moreover, evaluation findings provide practice-based evidence for decision making and ongoing program development or improvement. Because the Steps Program is time-bound, the use of evaluation findings during the program is a priority for many stakeholders.

### 3. Use the *Framework for Program Evaluation in Public Health* to inform evaluation planning.

The *Framework for Program Evaluation in Public Health (Framework) *is a practical, nonprescriptive tool designed to summarize and organize essential elements of program evaluation. The document (available from www.cdc.gov/eval/framework.htm)is a practical, nonprescriptive tool designed to summarize and organize essential elements of program evaluation. The document (available from www.cdc.gov/eval/framework.htm) recommends the following six steps for program evaluation: 1) engage stakeholders, 2) describe the program, 3) focus the evaluation design, 4) gather credible evidence, 5) justify conclusions, and 6) ensure use and share lessons learned (8). Each step includes subpoints that describe issues to consider when creating an evaluation plan (8). Another element of the *Framework* is a set of 30 standards for assessing the quality of evaluation activities, organized into four categories: utility, feasibility, propriety, and accuracy. Adhering to the steps and standards improves how public health practitioners conceive and conduct evaluation efforts. 

The *Framework* emphasizes the importance of constructing practical evaluation strategies that involve diverse program stakeholders, not just evaluation experts. For the Steps Program, attention to stakeholder roles throughout the process helps to ensure a participatory approach to program evaluation at the national, state, and community levels. Program evaluation is presented as ongoing and iterative, each step in the process informing the next; this approach is well suited to the context and complexity of community-based programs. Although the *Framework* provides a systematic process for program evaluation planning, it does not include all of the details of a formal plan. As such, the substance of the plan requires additional discussion and decision making with program stakeholders and staff.

Steps to a Healthier New York, which includes programs in Broome, Chautauqua, Jefferson, and Rockland counties, offers an example of stakeholder involvement. The New York State Department of Health coordinates program evaluation activities with staff in each of these communities. The group employs the *Framework *to plan and implement data collection intended to document program implementation and outcomes and identify opportunities for immediate and ongoing program improvement. The participation of national, state, and local stakeholders is the cornerstone of their approach. Stakeholder participation includes assessment of the composition of local consortia to ensure appropriate representation of varied stakeholders; regular site visits and assorted information-exchange opportunities; and formal agreements with local academic and health care institutions and others. Stakeholders in each of the communities provide knowledge of community context and characteristics to better frame programmatic, evaluation, and surveillance activities, resulting in an authentic definition of the program from a community perspective. Staff used this community-based definition to identify both evidence-based interventions and measurement strategies to meet local needs.

The evaluation design for Steps to a Healthier New York rests on the following four pillars defined by staff and stakeholders: 1) disease and risk factor surveillance via the BRFSS and YRBSS; 2) systematic program monitoring to assess implementation and early outcomes; 3) assessment of longer-term outcomes at the community level; and 4) strategic coordination with national-level evaluation activities. Stakeholders prioritized evaluation questions on the path to a complete evaluation and implementation plan based on these pillars. Thus, systems are in place to provide a steady stream of credible information for each community. The constant exchange of information among these communities led to practical and cost-effective methods for demonstrating progress toward program goals, identifying opportunities for program improvement, and sharing lessons learned to enhance chronic disease prevention and health promotion efforts statewide. For this program, the six steps and related standards have proven to be an effective approach to better integrating disease and risk factor surveillance, program, and evaluation planning with an emphasis on stakeholder participation across four distinct communities in New York.

### 4. Seek cultural competence in program evaluation planning, implementation, and use of findings.

Cultural competence in program evaluation practice is a theme throughout the *Framework.* However, the authors do not explicitly define cultural competence. As a starting point for practice across the Steps Program, cultural competence in program evaluation "involves a set of academic or interpersonal skills that allow individuals to increase their understanding and appreciation of cultural differences and similarities within, among, and between groups" (25). To demonstrate cultural competence, the public health practitioner must draw on community-based values, traditions, and customs and work with knowledgeable individuals from the community to develop focused interventions and communications (25). "Successful and explicit identification of stakeholders' values and interests is the bedrock of cultural competence in evaluation" (26).

To achieve cultural competence, program evaluation must be responsive to cultural context, use appropriate frameworks and methodology, and rely on "stakeholder-generated, interpretive means to arrive at the results and further use of findings" (11). To illustrate, Steps to a Healthier Anishinaabe spans 38 Michigan counties and serves the Bay Mills Indian Community, Grand Traverse Bands of Ottawa and Chippewa Indians, Hannahville Indian Community, Huron Potawatomi Indian Community, Keweenaw Bay Indian Community, Little Traverse Bay Band of Odawa Indians, Saginaw Chippewa Indian Tribe, and Sault Ste Marie Tribe of Chippewa Indians. The geographic distribution of participants across the state and stakeholder concerns about participation in the BRFSS resulted in development of an approach to disease and risk factor surveillance driven by community values.

The conventional approach to sampling for participation in the BRFSS was impractical for this program. Steps to a Healthier Anishinaabe includes sovereign tribes, each with a unique infrastructure. American Indian households can be difficult to identify for the purposes of creating a typical sampling frame. The release of enrollment data requires approval at the highest levels of tribal government, and many stakeholders consider distribution of this information counter to community values and priorities. As such, Michigan's Behavioral Risk Factor Survey does not already oversample this population.

In collaboration with each of the listed tribes listed, and with support from the Centers for Disease Control and Prevention's (CDC's) Steps Program Office (SPO) and Division of Adult and Community Health, the Inter-Tribal Council of Michigan (ITCM) developed a culturally appropriate strategy for participation in the BRFSS. The approach draws on localized definitions of culturally competent practice and related ethics to meet the information needs of decision makers at the national level and among tribes involved. For the majority of these tribes, inclusion in the sampling frame was actively voluntary. Tribal members submitted their phone numbers via boxes at each site. Tribes offered modest incentives for participation. The approach resulted in approximately 7800 phone numbers. The ITCM provided these numbers, without identifiers, for sampling and data collection. 

In this community, ongoing dialogue, respect for tribal values, and flexibility resulted in full participation in the data collection requirements of the Steps Program. Stakeholders at the national and community levels maximized an opportunity to improve surveillance and evaluation practice and ensure culturally competent service (i.e., data collection) to tribal members.

Although cultural competence may look different at the national and community levels and vary in concept and practice across the Steps Program, it is critical to the ultimate usefulness of an evaluation. Appropriate attention to cultural competence throughout the evaluation process reflects and affirms principles, ethics, and standards for program evaluation in public health. Moreover, it ensures a consequential role for diverse program stakeholders and compels greater use of the evaluation for decision making.

### 5. Prepare a program logic model as a platform for evaluation of planning, implementation, and use of findings.

A logic model is a picture of a program that shows the relationships among resources, activities, and the benefits, or changes, that result over time (16). Often referred to as *theory of change* (16), *program theory* (27), or *theory of action* (6), the graphic presentation is a "plausible, sensible model of how a program is supposed to work" (28). Specifically, "a theory of change is a description of how and why a set of activities — be they part of a highly focused program or a comprehensive initiative — are expected to lead to early, intermediate and longer term outcomes over a specified period" (16). Attention to and presentation of program theory is the distinguishing characteristic of a program logic model.

A basic logic model includes inputs, activities, outputs, and outcomes (short-term, intermediate, and long-term). Inputs include program resources (e.g., human and fiscal resources, organizational capacities, existing infrastructure). Outputs are the direct products of program activities (e.g., programs or services delivered, number of people served, work completed). Outcomes are the results, effects, or benefits of public health programs; they are the changes that occur for individuals, groups, families, households, organizations, or communities during or after the program (e.g., changes in behavior, norms, knowledge, attitudes, policy, capacities, and conditions). It is important to understand the difference between outputs and outcomes. Outputs relate to "what we do" as public health practitioners, and outcomes refer to "what difference is there" because of these efforts (16). Sound evidence (e.g., public health research, intervention science, practice-based knowledge) clarifies the relationships among the components of a logic model. However, the evidence base for programs may not be well developed or easily accessible. As such, discussions of program theory should include an appraisal of a broad range of sources of evidence to link program inputs with activities and outcomes. Furthermore, an evaluability assessment checks "whether or not a program is logically theorized, planned, and resourced" with the aim of avoiding investment in a program that was poorly designed (3). A well-designed logic model provides a platform for program and evaluation planning, program management, ongoing program development, and strategic communications.

### 6. Identify the purpose of the evaluation.

The purpose of the evaluation differs from the purpose of the program; articulating the purpose of the evaluation will “prevent premature decision making regarding how the evaluation should be conducted” (8). Program evaluation has at least four general purposes: 1) gain insight (e.g., document or assess an innovative approach to practice); 2) change practice (e.g., improve operations, refine program strategy, improve quality or efficiency); 3) assess effects (e.g., document program outcomes, intended and unintended); and 4) affect participants (e.g., serve as a catalyst for self-directed change among stakeholders, spur staff development, contribute to organizational change) (8). Moreover, the definition and pursuit of clear, appropriate purposes of the evaluation contributes to institutionalizing program evaluation within an organization (29).

An explicit statement of the purpose of the evaluation adds clarity and focus to the study and enhances usability among stakeholders with limited knowledge of program evaluation. With the purposes of the evaluation agreed upon, subsequent pieces of the study fall into place more easily (e.g., allocation of resources, identification of key evaluation questions, selection of appropriate sources of data). Stakeholders in the Steps Program delineated the following purposes of the national evaluation early in the planning process: assess the merit or worth of the Steps Program or key efforts; document program processes and progress toward intended outcomes; identify opportunities for ongoing program improvement; demonstrate accountability for resources to key stakeholders; and identify opportunities for ongoing program development and improvement.

### 7. Identify the intended users and uses of the evaluation.

The goal of utilization-focused evaluation is intended use by primary intended users (18). Primary users of the evaluation include stakeholders who are in a position to do or decide something about the program (8). Frequent interaction with primary users early in the evaluation process increases the likelihood that the evaluation will satisfy their information needs (6). "Use" refers to the application of information generated from the evaluation. However, lessons learned in the course of an evaluation do not automatically translate into decision making and action. Ongoing use of evaluation findings involves strategic thinking and continued vigilance from the earliest stages of stakeholder participation (8). All uses must be linked to one or more specific users to ensure that program resources are allocated to meet priority information needs. Explicit attention to intended use helps practitioners avoid "measurement mania" because only data that will be used for a specific purpose are collected.

### 8. Identify key evaluation questions.

The key to designing an evaluation that best meets stakeholder needs is precisely defining the questions that the study is expected to answer (30). Evaluation questions form the heart of the evaluation plan and pragmatic decisions about design and data collection methods. Evaluation questions stem from a shared understanding of the program's logic model and the defined purpose, users, and uses of an evaluation. Specific questions establish practical boundaries for the evaluation by defining exactly the facets of the program that will be addressed (6,18,27,31). Prioritizing questions among stakeholders further refines a focus for the evaluation and informs every one of the technical decisions to follow (e.g., identification of indicators, data collection methods, instrument design).

Often, evaluation questions are implicitly understood by program staff and consultants closest to the study and therefore are not included as an explicit component of the evaluation plan. However, a participatory approach to evaluation requires stakeholder involvement in the identification of evaluation questions. For the Steps Program, individuals responsible for program evaluation at the national, state, and community levels are encouraged to discuss, prioritize, and articulate key evaluation questions to ensure that evaluation practice and products are meaningful to all stakeholders.

### 9. Attend to process and outcome evaluation.

Public health programs should be evaluated in terms of their processes and outcomes. While outcome evaluation is used to assess whether a program works, it cannot typically demonstrate why or how the program works (or does not work). Knowledge of why or how a program creates change is as relevant in public health programs as information about whether a desired change occurred (2). Process evaluation is the systematic collection of information to document and assess program implementation and operations (5). This type of evaluation involves documentation and description of program activities — what, how much, for whom, when, and by whom (32). For example, process evaluation can be used to document the allocation and use of resources; assess recruitment, reach, or participation; determine "dose" delivered and received; and measure program fidelity (i.e., the extent to which the intervention was delivered as planned, quality of the intervention, and integrity of implementation) (20).

For example, Boston Steps includes programmatic activities in seven neighborhoods: Dorchester, Hyde Park, Jamaica Plain, Mattapan, Roxbury, South Boston, and South End/Chinatown. A cornerstone of the program, NeighborWalk, is an evidence-based community walking initiative. The Boston Public Health Commission, in collaboration with the Harvard Prevention Research Center, uses three tools to document implementation and participation. One, to document community participation in the program, walk coordinators citywide submit a weekly summary of activities (e.g., number of participants, steps walked as recorded on pedometers, duration of walks). Two, NeighborWalk participants volunteer to complete enrollment and exit questionnaires that capture demographic information about themselves and their relevant health-related behaviors. Three, walk coordinators complete brief narratives to document and describe perceived successes, barriers, and practical suggestions to improve NeighborWalk before the next cycle of activities. Each of the tools offers unique data for program monitoring and informed decision making (e.g., allocation of resources). This investment in process evaluation allows staff to quickly assess whether the program is reaching its intended audience, describe actual characteristics of the program in diverse neighborhoods, and identify improved pathways to influence health-related behaviors in these communities.

The definition and application of "process," "outcome," and "impact" evaluation vary in public health and beyond. PRECEDE–PROCEED, a prominent model for program planning, implementation, and evaluation in public health, positions impact evaluation to measure short-term effects defined as knowledge, skills, and behavior. Outcome evaluation measures health or quality of life (33). Yet, in many settings, impact evaluation refers to assessment of the most distal outcomes. To establish a common vocabulary and comparable practice for the Steps Program, outcome evaluation has been defined as the systematic collection of information about the results, effects, or benefits (intended and unintended) of programs during or after participation. To capture a range of outcomes as the Steps Program matures, program evaluation at the national and community levels includes both outcome and impact measurement through identification of short-term, intermediate, and long-term outcomes. Thus, for the purposes of common vocabulary and clarity, we do not reference impact evaluation from this point forward.

### 10. Maximize use of existing surveillance systems for outcome measurement.

Surveillance is the "ongoing, systematic collection, analysis, and interpretation of outcome-specific data for use in planning, implementing, and evaluating public health practice" (34). Monitoring of disease or risk factors through surveillance is a necessary component of comprehensive public health programs (34). In a climate of increased accountability for limited resources, programs realize certain efficiencies by using existing surveillance data for evaluation purposes (e.g., measuring progress toward intended outcomes). The use of these data greatly enhances consistency in measurement and comparability among programs.

The release of Indicators for Chronic Disease Surveillance (22) provides a comprehensive and recommended set of measures for chronic disease prevention and health promotion programs. The Steps Program uses relevant indicators to document progress toward intended outcomes; data sources for these indicators include BRFSS and YRBSS. Both surveillance systems can be used to determine the prevalence of health risk behaviors; assess whether health risk behaviors increase, decrease, or stay the same over time; examine the co-occurrence of health risk behaviors; provide comparable national, state, and local data; and monitor progress toward achieving the *Healthy People 2010* objectives and specific program outcomes. Whenever possible, the Steps Program uses data from these sources for national- and community-level program planning and evaluation purposes.

For example, Steps to a Healthier Colorado includes programs in Mesa, Pueblo, Teller, and Weld counties. The Colorado Department of Public Health and Environment coordinates surveillance and evaluation activities with staff in each of these communities. Program staff identified outcomes for measurement from three primary sources: *Healthy People 2010*, Indicators for Chronic Disease Surveillance, and community-specific selections drawn from local stakeholder priorities and needs. Steps to a Healthier Colorado enhanced existing surveillance systems to collect data in funded communities and ensure quality information to assess progress toward short-term, intermediate, and long-term outcomes. The BRFSS includes adults aged 18 years and older, the YRBSS includes youth in grades 9 through 12, and the state-based Child Health Survey includes children aged 1 to 14 years. These surveys provide data for both strategic program planning and the tracking of progress toward desired health outcomes. To maximize use for program evaluation, staff enhanced each survey to include additional short-term and intermediate measures relevant to community-based programming and objectives.

## Conclusion

Monitoring and evaluation are included among essential public health services as important components of efforts to promote continuous quality improvement of public health systems and related programs. The information presented here makes visible the overarching direction of evaluation practice across the Steps Program, including attention to the intended use of findings for accountability and continued program development. Foundational elements for program evaluation planning, implementation, and use of findings highlighted here illustrate the commitment of the Steps Program to improving public health, not only through service to communities but also through careful and appropriate documentation of program implementation and outcomes to provide practice-based evidence for decision making now and in the future.

## Figures and Tables

**Table:** Foundational Elements for Program Evaluation Planning, Implementation, and Use of Findings, Steps to a HealthierUS Cooperative Agreement Program

**Foundational Elements for Program Evaluation Planning, Implementation, and Use of Findings **	**Summary**	**Resources**
1. Distinguish between research and program evaluation.	Understanding differences between research and program evaluation encourages consideration of more options for the evaluation of public health programs. Research and program evaluation differ along 10 critical dimensions: planning, decision making, standards, questions, design, data collection, analysis and synthesis, judgments, conclusions, and uses.	Guidelines for defining public health research and public health non-research (4). MacDonald et al (5).
2. Define program evaluation.	The Steps Program defines program evaluation as "the systematic collection of information about the activities, characteristics, and outcomes of programs to make judgments about the program, improve program effectiveness, and/or inform decisions about future program development" (6).	Mathison (7). Patton (6).
3. Use the *Framework for Program Evaluation in Public Health* to inform evaluation planning.	The *Framework for Program Evaluation in Public Health *provides steps and standards for evaluation practice. The evaluation process is presented as ongoing, nonlinear, and participatory. Adhering to the steps and standards improves how public health practitioners conduct evaluations.	Framework for program evaluation in public health (8). Practical evaluation of public health programs workbook (9). Stufflebeam (10).
4. Seek cultural competence in program evaluation planning, implementation, and use of findings.	Program evaluation should be responsive to cultural context, use appropriate frameworks and methodology, and rely on "stakeholder-generated, interpretive means to arrive at the results and further use of findings" (11). Attention to cultural competence affirms principles, ethics, and standards for program evaluation.	Frierson et al (12). Thompson-Robinson et al (13).
5. Prepare a program logic model as a platform for evaluation planning, implementation, and use of findings.	A logic model makes visible the underlying theory of the program or intervention and connects resources invested with expected results. A logic model includes inputs, activities, outputs, and outcomes (short-term, intermediate, and long-term). A well-designed logic model guides program planning, evaluation, management, and communications.	McLaughlin and Jordan (14). Millar et al (15). Taylor-Powell et al (16).
6. Identify the purpose of the evaluation.	An explicit statement of the evaluation's purpose focuses and clarifies the planning process. After the purpose of the evaluation is agreed upon, subsequent decisions can be made more easily (e.g., allocation of resources, identification of evaluation questions, selection of data collection methods).	The program evaluation standards (17).
7. Identify intended users and uses of the evaluation.	Identification of intended users and uses is a necessary component of appropriate evaluation design. Users and uses must be prioritized so that resources for specific tasks can be allocated strategically.	Patton (18).
8. Identify key evaluation questions.	Evaluation questions follow from the stated purpose, users, and intended use of findings. Evaluation questions should be made explicit so that data collected meet the information needs of program stakeholders.	Frechtling and Sharp (19).
9. Attend to process and outcome evaluation.	Program processes are linked to outcomes by the theory of change presented in a program logic model. Outcome measures cannot demonstrate why or how a program works or does not work. Knowing why and how a program brings about desired outcomes is as important as knowing whether a desired outcome occurred.	Health promotion evaluation: recommendations to policy-makers (2). Starr et al (20). Steckler and Linnan (21).
10. Maximize use of existing surveillance systems for outcome measurement.	Evaluation of public health programs is often more efficient when existing surveillance data are used for outcome measurement. Use of these data enhances consistency in measurement and comparability among participating sites and relevant national estimates.	Indicators for chronic disease surveillance (22). Behavioral Risk Factor Surveillance System (BRFSS) (23). Youth Risk Behavior Surveillance System (YRBSS) (24).
